# Management Strategies for Disappearing Colorectal Liver Metastases After Systemic Chemotherapy: Long‐Term Outcomes and Preoperative Prediction of ‘True Complete Response’

**DOI:** 10.1002/ags3.70200

**Published:** 2026-02-16

**Authors:** Taihei Soma, Ryo Ashida, Takeshi Kawakami, Katsuhisa Ohgi, Mihoko Yamada, Shimpei Otsuka, Yoshiyasu Kato, Kentaro Yamazaki, Katsuhiko Uesaka, Teiichi Sugiura

**Affiliations:** ^1^ Division of Hepatobiliary‐Pancreatic Surgery Shizuoka Cancer Center Shizuoka Japan; ^2^ Division of Gastrointestinal Oncology Shizuoka Cancer Center Shizuoka Japan

**Keywords:** chemotherapy, disappearing colorectal liver metastases, hepatectomy, true complete response

## Abstract

**Background:**

Determining whether to resect disappearing liver metastases (DLMs) after chemotherapy for colorectal liver metastases (CRLMs) remains challenging.

**Methods:**

Patients who underwent hepatectomy after systemic chemotherapy for initially unresectable CRLMs were reviewed. True complete response (CR) was defined as either resected DLMs with pathological CR or unresected DLMs without local recurrence. Long‐term outcomes were compared between patients with and without resection of all DLMs.

**Results:**

Among 58 patients, 26 (44.8%) had DLMs, totaling 106 lesions. True CR was achieved in 70.8% of DLM lesions. Long‐term outcomes did not differ between patients with and without resection of all DLMs (median recurrence‐free survival, 11.1 vs. 7.6 months, *p* = 0.594; median surgical failure‐free survival, 11.1 vs. 17.2 months, *p* = 0.758; median overall survival, 43.6 vs. 53.6 months, *p* = 0.819). DLM lesions with true CR had smaller initial diameters than those without true CR (5 vs. 9 mm, *p* = 0.013).

**Conclusion:**

Regardless of whether all DLMs were resected, patients had acceptable long‐term outcomes. DLM lesions with a larger initial diameter before chemotherapy may warrant proactive intraoperative exploration for residual disease using contrast‐enhanced ultrasonography.

## Abstract 2

Patients who underwent hepatectomy after systemic chemotherapy for initially unresectable colorectal liver metastases (CRLMs) showed acceptable long‐term outcomes, regardless of whether all disappearing liver metastases (DLMs) were resected. DLM lesions with a larger initial diameter before chemotherapy may warrant proactive intraoperative exploration using contrast‐enhanced ultrasonography to detect residual disease.

## 1 Introduction

In the treatment of colorectal liver metastases (CRLMs), increased options for chemotherapy regimens and molecularly targeted drugs have increased the chance of hepatectomy after systemic chemotherapy for patients with initially unresectable CRLMs, contributing to improved prognosis [1, 2, 3]. On the other hand, effective modern systemic chemotherapy highlighted a new clinical problem of disappearing liver metastases (DLMs), which refers to complete shrinkage on cross‐sectional imaging after systemic chemotherapy. DLMs are reported to occur in up to 37% of patients who undergo hepatectomy after systemic chemotherapy for initially unresectable CRLMs [4, 5, 6].

To perform hepatectomy after systemic chemotherapy for initially unresectable CRLMs, technical difficulties due to advanced tumors and impairment of the liver function due to chemotherapy are often major challenges for liver surgeons. In addition, the treatment of DLMs presents a dilemma in determining whether all DLM lesions should be resected. Currently, there is no definitive consensus on treatment strategies for DLMs [7, 8]. As chemotherapy and imaging modalities for CRLMs have advanced remarkably over the years, the prevalence of DLMs and pathological complete response (PCR) rates vary widely depending on the age of previous reports [9, 10, 11, 12].

The present study was conducted to elucidate the postoperative outcomes of patients with DLMs, aiming to clarify the optimal treatment strategy for DLMs in the era of modern imaging and treatment modalities.

## 2 Methods

### 2.1 Patients

**FIGURE 1:**
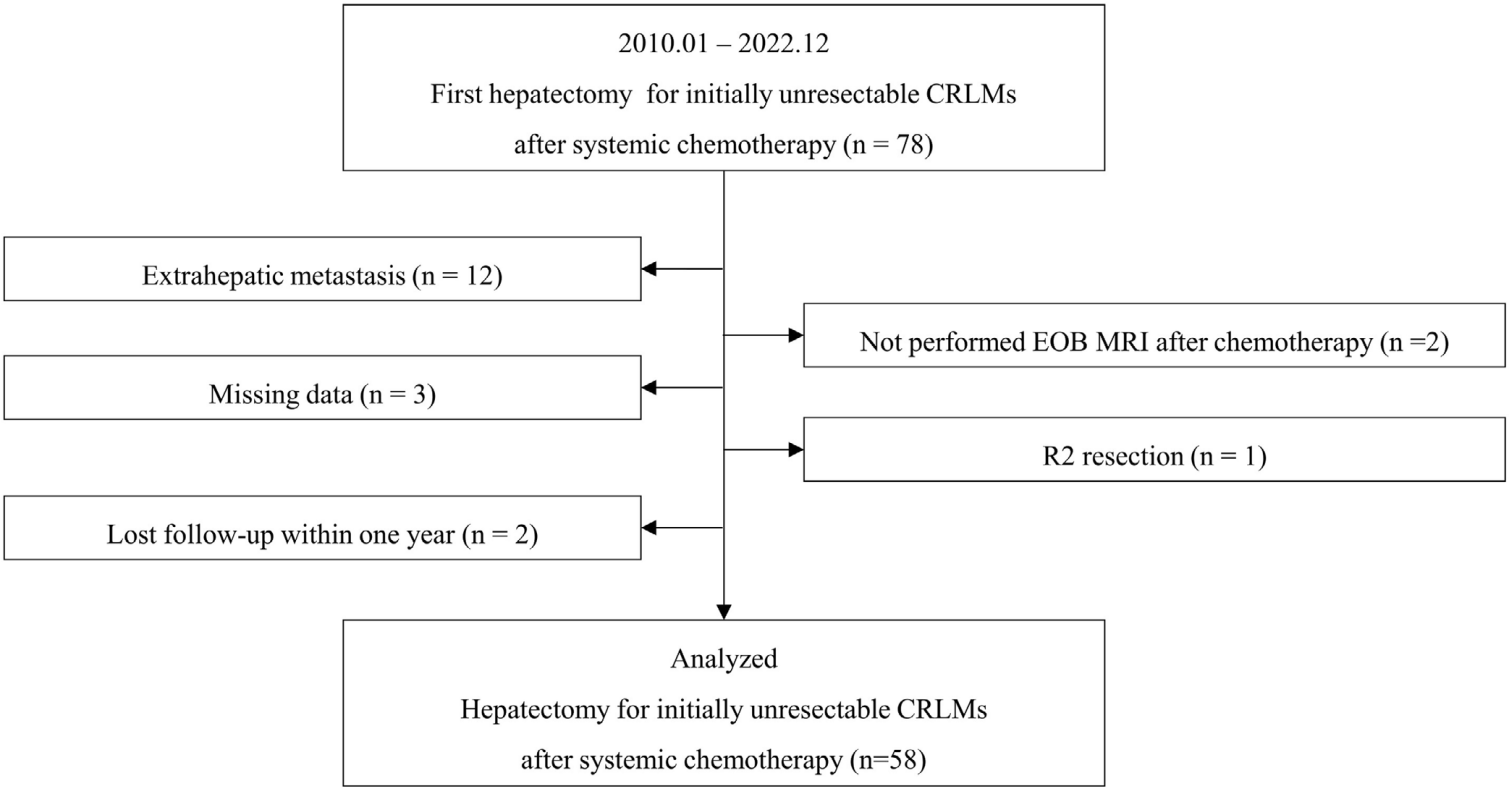
Patient selection. CRLMs, colorectal liver metastases; EOB‐MRI, gadoxetic acid‐enhanced magnetic resonance imaging.

Fifty‐eight consecutive patients who underwent hepatectomy after systemic chemotherapy for initially unresectable CRLMs at Shizuoka Cancer Center between January 2010 and December 2022 were retrospectively reviewed. The following exclusion criteria were applied: history of hepatectomy for CRLMs, any extrahepatic metastases, gadoxetic acid‐enhanced magnetic resonance imaging (EOB‐MRI) not performed before hepatectomy, insufficient clinical data, R2 resection, and loss to follow‐up within one year after hepatectomy (Figure 1).

This study was approved by the Institutional Review Board of Shizuoka Cancer Center (approval number J2023‐238‐2023‐1‐3).

### 2.2 Treatment Strategy for CRLMs

CRLMs were considered resectable if they met the following criteria: removal of all metastases with preservation of the liver parenchyma with blood inflow and outflow was considered technically possible and future liver remnant indocyanine green (ICG) K ≥ 0.05. The number of CRLMs was not a limiting factor [13, 14]. Borderline resectable (BR) CRLMs was defined as technically resectable but oncologically unfavorable tumors, such as marked increase in number among short period. Unresectable (UR) CRLMs was defined as technically unresectable tumors, such as insufficient future remnant liver volume.

Resectable CRLMs was resected without neoadjuvant chemotherapy. For BR‐CRLMs, systemic chemotherapy was initiated and considered to be indicated for hepatectomy if there was no progression or no elevation in tumor markers during chemotherapy. For UR‐CRLMs, systemic chemotherapy was performed as well and hepatectomy was planned if it became technically resectable with sufficient future remnant liver function after tumor shrinkage or separation from major vessels. Radiofrequency ablation was not adopted as a standard treatment modality in our institution. All patients initiated treatment after receiving approval from a multidisciplinary team conference.

In principle, contrast‐enhanced computed tomography (CE‐CT) and EOB‐MRI were performed before and after chemotherapy [15]. Contrast‐enhanced ultrasonography was not routinely performed.

### 2.3 Definition of DLMs and “True CR”

DLMs were defined as CRLM lesions that presented on initial imaging and that were no longer visible on EOB‐MRI after systemic chemotherapy [10, 16]. As an indicator of favorable prognosis, we also evaluated the rate of true complete response (true CR), which was defined as resected DLM lesions without viable tumor on pathological examination or unresected DLMs without local recurrence during at least one year of follow‐up [17].

### 2.4 Surgical Procedure

Parenchyma‐sparing hepatectomy is the standard treatment strategy. Liver dissection was performed using ultrasonic devices or a crush‐clamp procedure. The Pringle maneuver was performed. Intraoperative ultrasonography (IOUS) was routinely performed to confirm the preoperative diagnosis and identify new lesions. Contrast‐enhanced IOUS (CE‐IOUS) was performed if necessary [18].

DLMs were resected if lesions on the surface were visually recognized or if lesions inside the liver parenchyma were detected on IOUS. In cases where DLMs were not identified, blind hepatectomy was considered to perform in accordance with anatomic landmarks on preoperative imaging if the remnant liver volume is preserved and resection is limited to less than a sectionectomy.

### 2.5 Comparison of Long‐Term Outcomes

As long‐term survival outcomes, in addition to overall survival (OS) and recurrence‐free survival (RFS), surgical failure‐free survival (SFFS: defined as the time until unresectable relapse or death) [19] was evaluated as a complementary surrogate endpoint and compared between patients in whom all DLMs were resected and those in whom any DLMs were left unresected. These endpoints were calculated from the date of hepatectomy.

OS was compared between patients who achieved true CR and those who did not, to assess the association between true CR and prognosis.

### 2.6 Statistical Analysis

Categorical variables were compared using the chi‐square test or Fisher's exact test, as appropriate. Continuous variables were compared using the Mann–Whitney *U* test. Long‐term outcomes were calculated using the Kaplan–Meier method, and statistical differences were examined using the log‐rank test. The receiver operating characteristic (ROC) curve was created by plotting the sensitivity against the false‐positive rate (1‐specificity) at various threshold settings. We utilized the area under the curve (AUC) value and the Youden index [20]. Statistical significance was set at *p* < 0.05. All statistical analyses were conducted using EZR (Saitama Medical Center, Jichi Medical University, Saitama, Japan) [21].

## 3 Results

### 3.1 Patient Characteristics

Of the 58 patients, 4 did not undergo EOB‐MRI before chemotherapy for reasons including treatment at other institutions, whereas all other patients underwent CE‐CT and EOB‐MRI both before and after chemotherapy. Table 1 shows the baseline characteristics, details of tumors, systemic chemotherapy, hepatectomy, and clinical courses after hepatectomy in the patients who underwent hepatectomy for initially unresectable CRLMs after systemic chemotherapy. Among 58 patients, 26 patients (44.8%) had a total of 106 DLM lesions after systemic chemotherapy. There were no significant differences in the baseline patient characteristics between patients who had DLMs and those who did not. Patients with DLMs had a higher number of CRLMs and smaller maximum diameter before chemotherapy in comparison to patients without DLMs. The median follow‐up period of the censored cases after hepatectomy was 43 (range, 15–139) months.

**TABLE 1:** Characteristics of 58 patients who underwent hepatectomy for initially unresectable CRLMs after systemic chemotherapy. Comparison according to the presence of DLMs.

	DLMs (−) *n* = 32	DLM (+) *n* = 26	*p*
**Patient**			
Age (years)^a^	63 (43–78)	66 (42–78)	0.175
Sex (M/F)	22/10	19/7	0.778
BMI (kg/m^2^)^a^	22.3 (15.8–33.1)	22.6 (17.6–33.6)	0.900
CEA before chemotherapy (U/mL)^a^	118 (2–10 027)	81 (2–5976)	0.335
CEA before hepatectomy (U/mL)^a^	5 (1–687)	4 (1–1598)	0.784
**Primary tumor**			
Location (right colon/left colon/rectum)	10/9/13	3/9/14	0.210
Depth (T1/T2/T3/T4)	0/1/18/12	0/2/12/11	0.617
Histological type (tub1/tub2/por)	5/25/1	4/18/3	0.499
Vanous invasion (negative/positive)	10/18	7/18	0.733
Lymphatic invasion (negative/positive)	14/14	12/13	0.520
Perineural invasion (negative/positive)	9/12	10/11	1.000
LN metastases (negative/positive)	12/20	7/19	0.417
Lateral LN metastases (negative/positive)	0/13	1/13	1.000
KRAS mutation (negative/positive)	16/5	19/6	1.000
BRAF mutation (negative/positive)	11/0	14/0	N/A
**CRLMs**			
Synchronous/metachronous	28/4	24/2	0.681
Reason for unresectable (BR/UR)	12/20	11/15	0.790
Number before chemotherapy^a^	7 (1–17)	10 (2–23)	0.005
Number before hepatectomy^a^	7 (1–28)	7 (1–19)	0.882
Maximum diameter before chemotherapy^a^	68 (14–140)	39 (10–75)	0.009
Maximum diameter before hepatectomy^a^	32 (7–128)	17 (5–69)	0.013
Number of DLMs (if present)^a^	—	4 (1–12)	—
**Systemic chemotherapy**			
1st line (FOLFOX/XELOX/FOLFIRI/FOLFOXIRI)	21/5/3/3	17/2/3/4	0.799
Molecular targeted agent (yes/no)	31/1	25/1	1.000
Number of cycles^a^	9 (1–25)	10 (3–34)	0.252
**Hepatectomy**			
Open/MIS	32/0	25/1	0.448
Major^b^/Minor	22/10	14/12	0.285
ICG R15 (%)	11.7 (3.4–26.0)	11.0 (2.7–23.4)	0.532
Estimated resection rate (%)	51.6 (11.5–68.7)	49.4 (35.0–62.7)	0.808
**Clinical course after hepatectomy**			
Adjuvant chemotherapy after hepatectomy (yes/no)	11/21	7/19	0.581
Recurrence after hepatectomy (yes/no)	23/9	20/6	0.767
First recurrence site (DLM site/remnant liver/other)	−/12/15	5/5/15	N/A

Abbreviations: BR, borderline resectable; BRAF, B‐Raf proto‐oncogene, serine/threonine kinase; cape‐OX, capecitabine+L‐OHP; CEA, carcinoembryonic antigen; CR, complete response; CRLMs, colorectal liver metastases; DLMs, disappearing liver metastases; F, female; FOLFIRI, 5‐FU + LV + CPT‐11; FOLFOX, 5‐FU + LV + L‐OHP; FOLFOXIRI, 5‐FU + LV + L‐OHP + CPT; ICG, indocyanine green; KRAS, Kirsten rat sarcoma viral oncogene homolog; LN, lymph node; M, male; MIS, minimum invasive surgery; UR, unresectable.

^a^ Median.

^b^ Major hepatectomy was defined as removal of three or more Couinaud segments.

### 3.2 Details of Clinical Course in Patients With DLMs (Verification for Each Patient)

**FIGURE 2:**
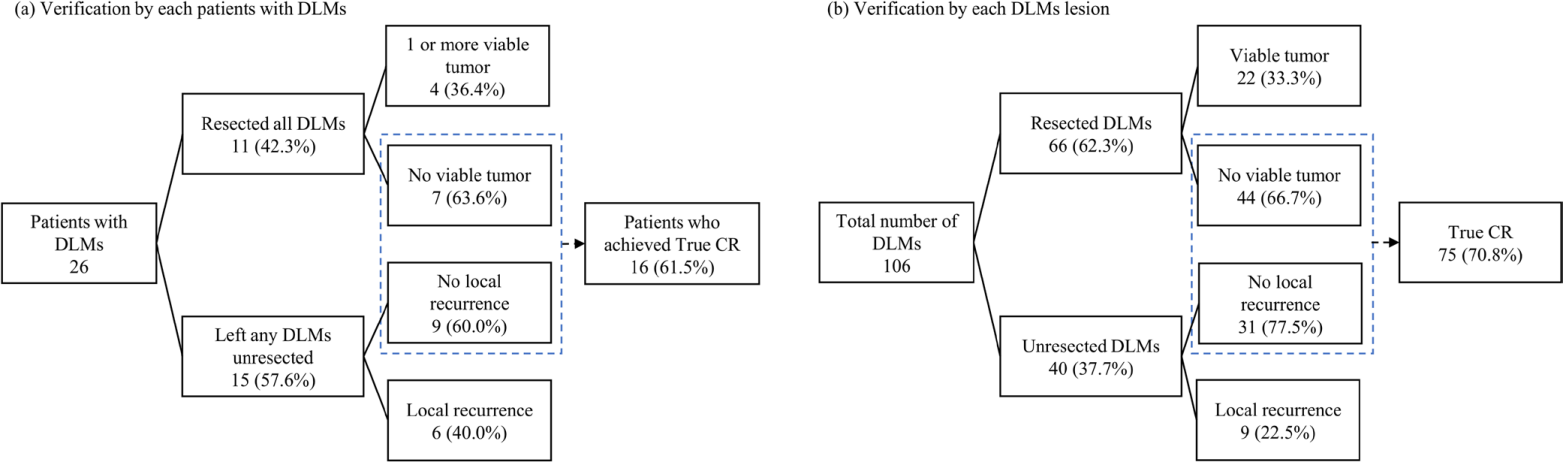
Details of DLMs. Management, follow‐up, and true CR rate. (a) Verification by each patient with DLMs. (b) Verification by each DLM lesion. DLMs, disappearing liver metastases: CR, complete response.

Figure 2a shows the details of the clinical course of the 26 patients with DLMs. All DLMs were resected in 11 patients. Any DLMs were left unresected in the remaining 15 patients. Among the 11 patients in whom all DLMs were resected, 7 (63.6%) achieved PCR, and the remaining 4 (36.4%) had at least one viable tumor. During follow‐up, among 15 patients in whom any DLMs were left unresected, 6 patients had recurrence at the site of an unresected DLM (median interval from hepatectomy to local recurrence: 4 [1, 2, 3, 4, 5, 6, 7, 8, 9, 10, 11, 12, 13] months). In summary, among the 26 patients with DLMs, 16 (61.5%) achieved a true CR.

Table 2 summarizes comparison of the baseline characteristics, details of tumors and clinical courses in 26 patients with DLMs, stratified by whether all DLMs were resected. No significant differences were observed in the baseline characteristics between patients in whom all DLMs were resected and those in whom they were not.

**TABLE 2:** Characteristics of 26 patients with DLMs after systemic chemotherapy for initially unresectable CRLMs. Comparison according to resect all DLMs or not.

	All DLMs resected *n* = 11	Any DLMs unresected *n* = 15	*p*
**Patient**			
Age (years)^a^	68 (59–74)	61 (42–78)	0.057
Sex (M/F)	8/3	11/4	1.000
BMI (kg/m^2^)^a^	23.1 (17.6–25.9)	21.9 (18.6–33.6)	0.876
CEA before chemotherapy (U/mL)^a^	27 (2–933)	88 (3–5976)	0.284
CEA before hepatectomy (U/mL)^a^	5 (3–51)	4 (1–1598)	0.203
**Primary tumor**			
Location (right colon/left colon/rectum)	2/4/5	1/5/9	0.741
Depth (T1/T2/T3/T4)	0/1/5/5	0/1/7/6	1.000
Histological type (tub1/tub2/por)	2/7/2	2/11/1	0.692
Vanous invasion (negative/positive)	3/8	4/10	0.197
Lymphatic invasion (negative/positive)	6/5	6/8	0.693
Perineural invasion (negative/positive)	6/5	4/6	0.670
LN metastases (negative/positive)	4/7	3/12	0.407
Lateral LN metastases (negative/positive)	5/0	6/1	1.000
KRAS mutation (negative/positive)	8/3	11/3	1.000
BRAF mutation (negative/positive)	9/0	5/0	N/A
**CRLMs**			
Synchronous/metachronous	11/0	13/2	0.492
Reason for unresectable (BR/UR)	6/5	5/10	0.426
Number before chemotherapy^a^	8 (2–19)	13 (3–23)	0.275
Number before hepatectomy^a^	6 (1–11)	9 (1–19)	0.212
Maximum diameter before chemotherapy^a^	37 (10–70)	40 (15–75)	0.716
Maximum diameter before hepatectomy^a^	19 (5–39)	15 (9–69)	0.533
Number of DLMs (if present)^a^	3 (1–6)	4 (1–12)	0.142
**Systemic chemotherapy**			
1st line (FOLFOX/XELOX/FOLFIRI/FOLFOXIRI)	8/1/1/1	9/1/2/3	0.909
Molecular targeted agent (yes/no)	11/0	14/1	1.000
Number of cycles^a^	7 (3–14)	13 (6–34)	0.057
**Hepatectomy**			
Open/MIS	11/0	14/1	1.000
Major^b^ /Minor	8/3	6/9	0.130
ICG R15 (%)	13.4 (2.7–23.4)	10.0 (3.7–21.0)	0.697
Estimated resection rate (%)	54.5 (39.0–62.7)	43.8 (35.0–57.2)	0.034
**Clinical course after hepatectomy**			
Adjuvant chemotherapy after hepatectomy (yes/no)	5/6	2/13	0.095
Recurrence after hepatectomy (yes/no)	8/3	12/3	0.407
First recurrence site (DLM site/remnant liver/other)	−/3/7	5/2/8	N/A

Abbreviations: BR, borderline resectable; BRAF, B‐Raf proto‐oncogene, serine/threonine kinase; CEA, carcinoembryonic antigen; CR, complete response; CRLMs, colorectal liver metastases; DLMs, disappearing liver metastases; F, female; FOLFIRI, 5‐FU + LV + CPT‐11; FOLFOX, 5‐FU + LV + L‐OHP; cape‐OX, capecitabine+L‐OHP; FOLFOXIRI, 5‐FU + LV + L‐OHP + CPT; ICG, indocyanine green; KRAS, Kirsten rat sarcoma viral oncogene homolog; LN, lymph node; M, male; MIS, minimum invasive surgery; UR, unresectable.

^a^ Median.

^b^ Major hepatectomy was defined as removal of three or more Couinaud segments.

### 3.3 Details of DLMs and Rate of True CR (Verification for Each DLM Lesion)

Figure 2b shows the verification for each DLM lesion. Among the 106 DLM lesions, 66 lesions were resected and the remaining 40 were left unresected. Of the 66 resected DLM lesions, residual microscopic viable tumors were found on histopathological examination in 22 (33.3%) lesions, and PCR was achieved in the remaining 44 lesions (66.7%). Of the 40 unresected DLM lesions, local recurrence occurred in 9 (22.5%), while the remaining 31 (77.5%) showed no recurrence. In summary, true CR was achieved in 75 of the 106 lesions (70.8%).

### 3.4 Comparison of Long‐Term Outcomes

**FIGURE 3:**
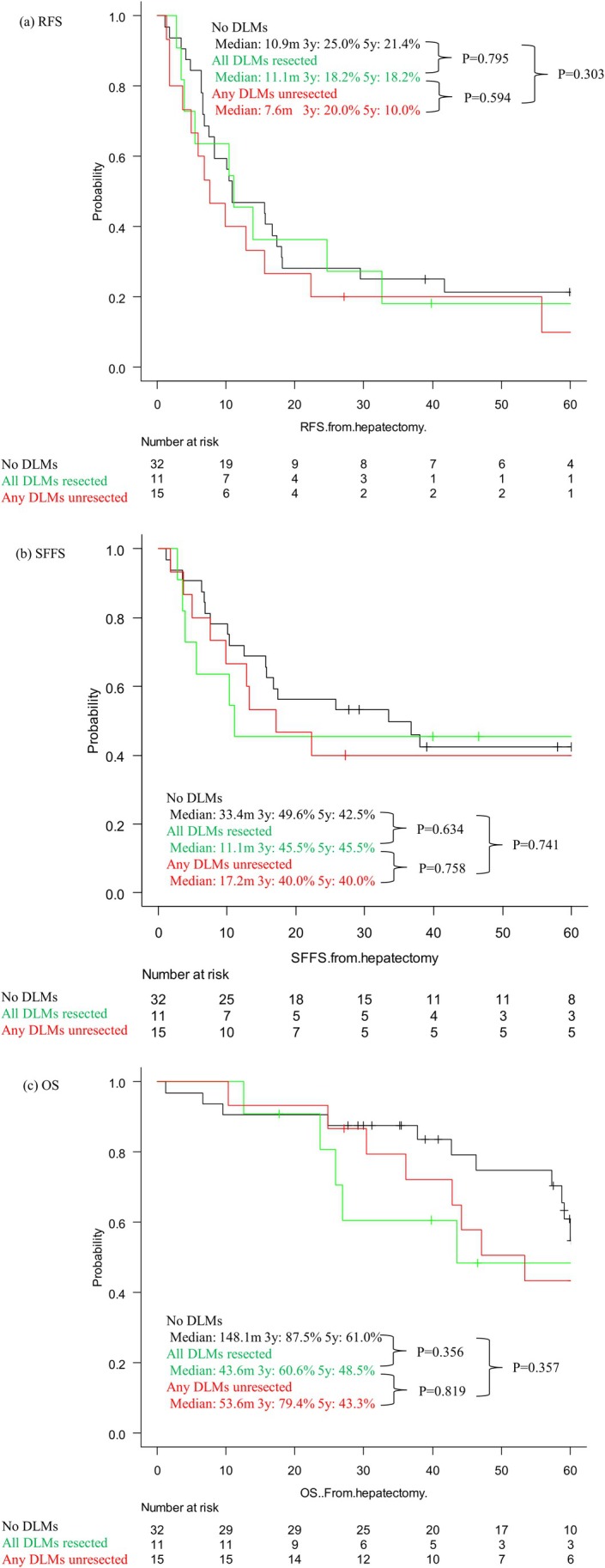
Comparison of (a) RFS, (b) SFFS and (c) OS among the following three groups: Patients with no DLMs, patients with all DLMs resected, and patients with any DLMs unresected. RFS, recurrence‐free survival; SFFS, surgical failure‐free survival; OS, overall survival; DLMs, disappearing liver metastases.

Figure 3 shows a comparison of long‐term outcomes based on whether all DLMs were resected.

RFS (Figure 3a), SFFS (Figure 3b), and OS (Figure 3c) did not differ significantly among the three groups: patients with no DLMs, patients in whom all DLMs were resected, and patients with any unresected DLMs. The median RFS was 10.9, 11.1, and 7.6 months, respectively; the median SFFS was 33.4, 11.1, and 17.2 months, respectively; and the median OS was 148.1 months, 43.6, and 53.6 months, respectively.

To assess the impact of adjuvant chemotherapy after hepatectomy, long‐term outcomes were compared according to whether all DLMs were resected within the subgroup that did not receive adjuvant chemotherapy (Figure S1). The results were consistent with those observed in the overall cohort, with no significant differences between the two groups.

OS stratified by the achievement of true CR is also shown for the entire cohort (Figure S2a), the group with all DLMs resected (Figure S2b), and the group with any DLMs unresected (Figure S2c).

### 3.5 Preoperative Prediction of True CR

To identify predictors of true CR achievement, baseline characteristics and details of the primary disease and CRLMs were compared (Table 3) between patients who achieved true CR and those who did not. Although carcinoembryonic antigen (CEA) before systemic chemotherapy was tended to be lower in patients who achieved true CR, all parameters were comparable between the two groups.

**TABLE 3:** Baseline characteristics of 26 patients with DLMs after systemic chemotherapy for initially unresectable CRLMs. Comparison according to achieve true CR or not.

	Achieved true CR *n* = 16	Not achieved true CR *n* = 10	*p*
**Patient**			
Age (years)^a^	67 (48–72)	64 (42–78)	0.442
Sex (M/F)	12/4	7/3	1.000
BMI (kg/m^2^)^a^	23.0 (17.6–27.1)	22.3 (18.1–33.6)	0.979
CEA before chemotherapy (U/mL)^a^	55 (2–932)	85 (3–5976)	0.231
CEA before hepatectomy (U/mL)^a^	4 (2–1598)	4 (1–452)	0.732
**Primary tumor**			
Location (right colon/left colon/rectum)	2/7/7	1/2/7	0.406
Depth (T1/T2/T3/T4)	2/8/6	0/4/5	0.584
Histological type (tub1/tub2/por)	4/10/2	0/8/1	0.417
Vanous invasion (negative/positive)	4/11	3/7	1.000
Lymphatic invasion (negative/positive)	7/8	5/5	0.570
Perineural invasion (negative/positive)	7/6	3/5	0.659
LN metastases (negative/positive)	5/11	2/8	0.668
Lateral LN metastases (negative/positive)	7/0	4/1	0.417
KRAS mutation (negative/positive)	11/4	8/2	1.000
BRAF mutation (negative/positive)	9/0	5/0	N/A
**CRLMs**			
Synchronous/metachronous	15/1	9/1	1.000
Reason for unresectable (BR/UR)	6/10	5/5	0.689
Number before chemotherapy^a^	15 (3–23)	9 (2–22)	0.256
Number before hepatectomy^a^	8 (1–14)	6 (1–19)	0.303
Maximum diameter before chemotherapy^a^	37 (15–69)	54 (10–75)	0.304
Maximum diameter before hepatectomy^a^	17 (7–65)	20 (5–69)	0.460
Number of DLMs (if present)^a^	4 (1–12)	3 (1–6)	0.439
**Systemic chemotherapy**			
1st line (FOLFOX/XELOX/FOLFIRI/FOLFOXIRI)	9/2/3/2	8/0/0/2	0.359
Molecular targeted agent (yes/no)	15/1	10/0	1.000
Number of cycles^a^	9 (3–20)	12 (5–34)	0.404
**Hepatectomy**			
Open/MIS	11/5	9/1	0.352
Major^b^ /Minor	8/8	4/6	0.701
ICG R15 (%)	11.0 (2.7–17.0)	11.7 (5.6–23.4)	0.593
Estimated resection rate (%)	49.4 (35.0–60.5)	48.7 (36.8–62.7)	0.804
**Clinical course after hepatectomy**			
Adjuvant chemotherapy after hepatectomy (yes/no)	6/10	1/9	0.190
Recurrence after hepatectomy (yes/no)	10/6	9/1	0.190
First recurrence site (DLM site/remnant liver/other)	−/3/10	5/2/5	N/A

Abbreviations: BR, borderline resectable; BRAF, B‐Raf proto‐oncogene, serine/threonine kinase; cape‐OX, capecitabine+L‐OHP; CEA, carcinoembryonic antigen; CR, complete response; CRLMs, colorectal liver metastases; DLMs, disappearing liver metastases; F, female; FOLFIRI, 5‐FU + LV + CPT‐11; FOLFOX, 5‐FU + LV + L‐OHP; FOLFOXIRI, 5‐FU + LV + L‐OHP + CPT; ICG, indocyanine green; KRAS, Kirsten rat sarcoma viral oncogene homolog; LN, lymph node; M, male; MIS, minimum invasive surgery; UR, unresectable.

^a^ Median.

^b^ Major hepatectomy was defined as removal of three or more Couinaud segments.

**FIGURE 4:**
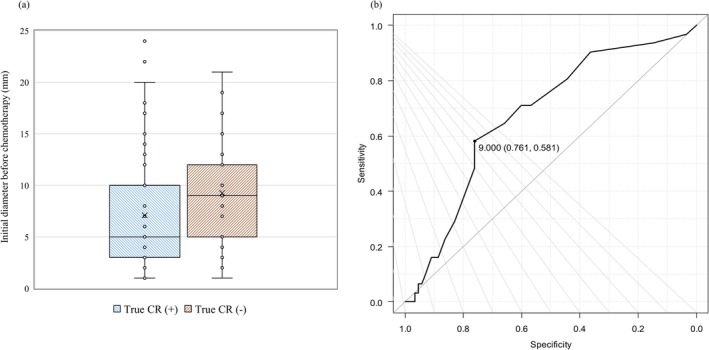
(a) Box plot of the initial diameter of CRLMs according to the achievement of true CR. (b) The ROC curve for the initial diameter of CRLMs in predicting the achievement of true CR. The AUC was 0.677 (95% CI, 0.569–0.784). The optimal cutoff for the initial diameter of the CRLM was 9 mm (sensitivity, 0.581; specificity, 0.761). CR, complete response; ROC, receiver operating characteristic; AUC, area under the curve; CI, Confidence Interval.

On the other hand, in a lesion‐by‐lesion analysis, lesions achieving true CR had a smaller initial tumor diameter before chemotherapy than those not achieving true CR (Figure 4a). The median initial tumor diameter before systemic chemotherapy was 5 mm in lesions achieving true CR and 9 mm in those not achieving true CR (*p* = 0.013). Figure 4b displays the ROC curve for the initial diameter of CRLMs in predicting the achievement of true CR, with an AUC of 0.677 (95% CI: 0.569–0.784). The optimal cutoff value for the initial diameter of the CRLMs was 9 mm (sensitivity, 0.581; specificity, 0.761). DLM lesions with an initial diameter of < 9 mm had a significantly higher true CR rate than those with an initial diameter of ≥ 9 mm (80.8% vs. 52.6%, *p* = 0.004).

## 4 Discussion

In the present study, we reviewed the prevalence and treatment outcomes of patients with DLMs in the modern era, when EOB‐MRI is the standard diagnostic modality, and when doublet, triplet chemotherapy regimens, and molecular targeted drugs are standard therapies for CRLMs. DLMs were observed in 42.3% of patients after systemic chemotherapy for initially unresectable CRLMs. True CR rates were 61.5% per patient and 70.8% per lesion. Long‐term outcomes were comparable between patients who underwent resection of all DLMs and those who did not.

A major value of this study is the comparison of long‐term outcomes of DLMs according to treatment strategies. In the present study, the impact of the treatment strategy for DLMs—whether to resect all DLMs or leave any DLMs unresected—on long‐term outcomes was not demonstrated. This finding is consistent with those of a Japanese single‐center prospective study [22] and an international multicenter prospective study [23], and further strengthen the existing evidence. This study also revealed that resection of all DLMs had only limited impact on long‐term outcomes and on the control of unresected DLM lesions in patients who did not receive adjuvant chemotherapy.

It is known that CRLMs often recur after hepatectomy [23, 24, 25]. In fact, as shown in this study, approximately 80% of patients had recurrence after any hepatectomy, and the sites of recurrence were not limited to unresected DLM lesions, recurrence was also observed in the remnant liver and other organs. This result may suggest that resecting all DLM lesions during hepatectomy has less of an impact on OS. It is preferable to perform resection to a sufficient extent and plan hepatectomy at the time of recurrence, rather than resecting undetectable DLMs by ‘blind hepatectomy’ and impairing the remnant liver function. However, it should be noted that the median time to recurrence at the site of unresected DLMs was relatively short, at four months. While the Japanese Society for Cancer of the Colon and Rectum guidelines recommend imaging surveillance every six months postoperatively [26], a more intensive follow‐up strategy using EOB‐MRI may be warranted [7].

Another important contribution of this study was preoperative prediction of true CR. The true CR rate per DLM lesion in this study was 70.8%, which was higher than the previously reported rate (38.9%–63.5%) [17, 27]. We showed that the initial diameter before systemic chemotherapy was useful for predicting the achievement of true CR. DLM lesions with larger initial diameters warrant special attention, as they are more likely to contain residual tumor even after shrinking radiographically. Although we agree that a watch‐and‐wait strategy may be generally appropriate for DLMs, in selected cases where the lesions had a larger initial diameter before chemotherapy, active exploration using intraoperative CE‐US may be beneficial in guiding consideration of surgical resection.

The present study has several limitations. First, the BR classification focuses solely on the number of liver metastases and does not account for tumor marker or tumor diameter [28, 29]. Therefore, the indications for preoperative chemotherapy at our institution may differ from those at other centers. Second, CE‐IOUS was not routinely used. Consequently, tumor identification during surgery may have been suboptimal [9, 25, 30], and it remains unclear whether all resected DLMs could be completely excised for microscopic evaluation, particularly in cases of blind hepatectomy. Third, important pathological and molecular factors with known prognostic significance after hepatectomy for colorectal liver metastases, such as KRAS (Kirsten rat sarcoma viral oncogene homolog) and BRAF (B‐Raf proto‐oncogene) mutations [31] and perineural invasion of the primary tumor [32] were not sufficiently evaluated because of substantial missing data.

In conclusion, regardless of whether all DLMs were resected under modern diagnostic techniques, patients who underwent hepatectomy after systemic chemotherapy for initially unresectable CRLMs had favorable long‐term outcomes. DLMs with a larger initial diameter before chemotherapy may have a higher likelihood of residual disease and therefore may warrant proactive exploration using intraoperative CE‐US.

## Author Contributions

**Taihei Soma:** conceptualization, methodology, data curation, investigation, validation, formal analysis, writing – original draft, project administration, writing – review and editing. **Ryo Ashida:** methodology, conceptualization, investigation, data curation, formal analysis, supervision, project administration, writing – review and editing, writing – original draft. **Takeshi Kawakami:** supervision, writing – review and editing. **Katsuhisa Ohgi:** supervision, writing – review and editing. **Mihoko Yamada:** supervision, writing – review and editing. **Shimpei Otsuka:** supervision, writing – review and editing. **Yoshiyasu Kato:** supervision, writing – review and editing. **Kentaro Yamazaki:** supervision, writing – review and editing. **Katsuhiko Uesaka:** supervision, writing – review and editing. **Teiichi Sugiura:** supervision, writing – review and editing.

## Funding

The authors have nothing to report.

## Ethics Statement

The study protocol for this research project was approved by a suitable Institutional Ethics Committee and conformed to the provisions of the Declaration of Helsinki. The Institutional Review Board of Shizuoka Cancer Center approved this study (Approval No. J2023‐238‐2023‐1‐3).

## Conflicts of Interest

The authors declare no conflicts of interest.

## Supporting information


**Supplemental FIGURE 1.** Subgroup analysis in patients who did not receive adjuvant chemotherapy after hepatectomy. Comparison of (a) RFS, (b) SFFS and (c) OS among the following three groups: patients with no DLMs, patients with all DLMs resected, and patients with any DLMs unresected.
RFS, recurrence‐free survival; SFFS, surgical failure‐free survival; OS, overall survival; DLMs, disappearing liver metastases.

**Supplemental FIGURE 2.** Comparison of OS according to whether a true CR was achieved.
OS was compared among: (a) 26 patients who had DLMs after systemic chemotherapy for initially unresectable CRLMs, (b) 11 patients in whom all DLMs were resected, and (c) 15 patients in whom any DLMs were left unresected.
OS, overall survival; CR, complete response; DLMs, disappearing liver metastases; CRLMs, colorectal liver metastases.
