# Supplementary figures and images for: Management Strategies for Disappearing Colorectal Liver Metastases After Systemic Chemotherapy: Long‐Term Outcomes and Preoperative Prediction of ‘True Complete Response’
Source: Ann Gastroenterol Surg. 2026 Feb 16;10(4):1239–49. doi: 10.1002/ags3.70200 (PMC13327054; doi:10.1002/ags3.70200)

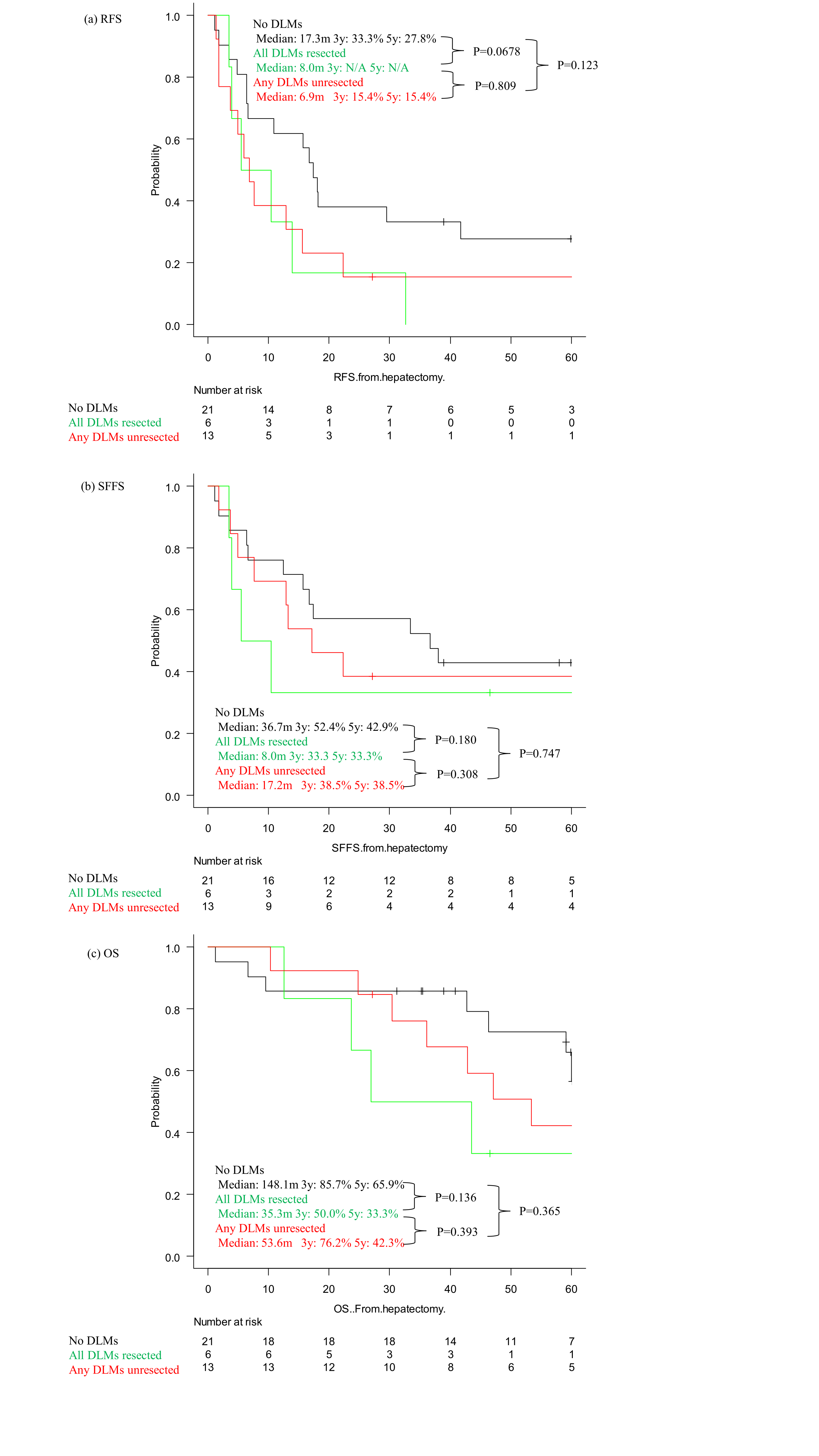

Supplement: Supplementary file 1 — Supplemental FIGURE 1. Subgroup analysis in patients who did not receive adjuvant chemotherapy after hepatectomy. Comparison of (a) RFS, (b) SFFS and (c) OS among the following three groups: patients with no DLMs, patients with all DLMs resected, and patients with any DLMs unresected. RFS, recurrence‐free survival; SFFS, surgical failure‐free survival; OS, overall survival; DLMs, disappearing liver metastases. [file AGS3-10-1239-s001.tif]

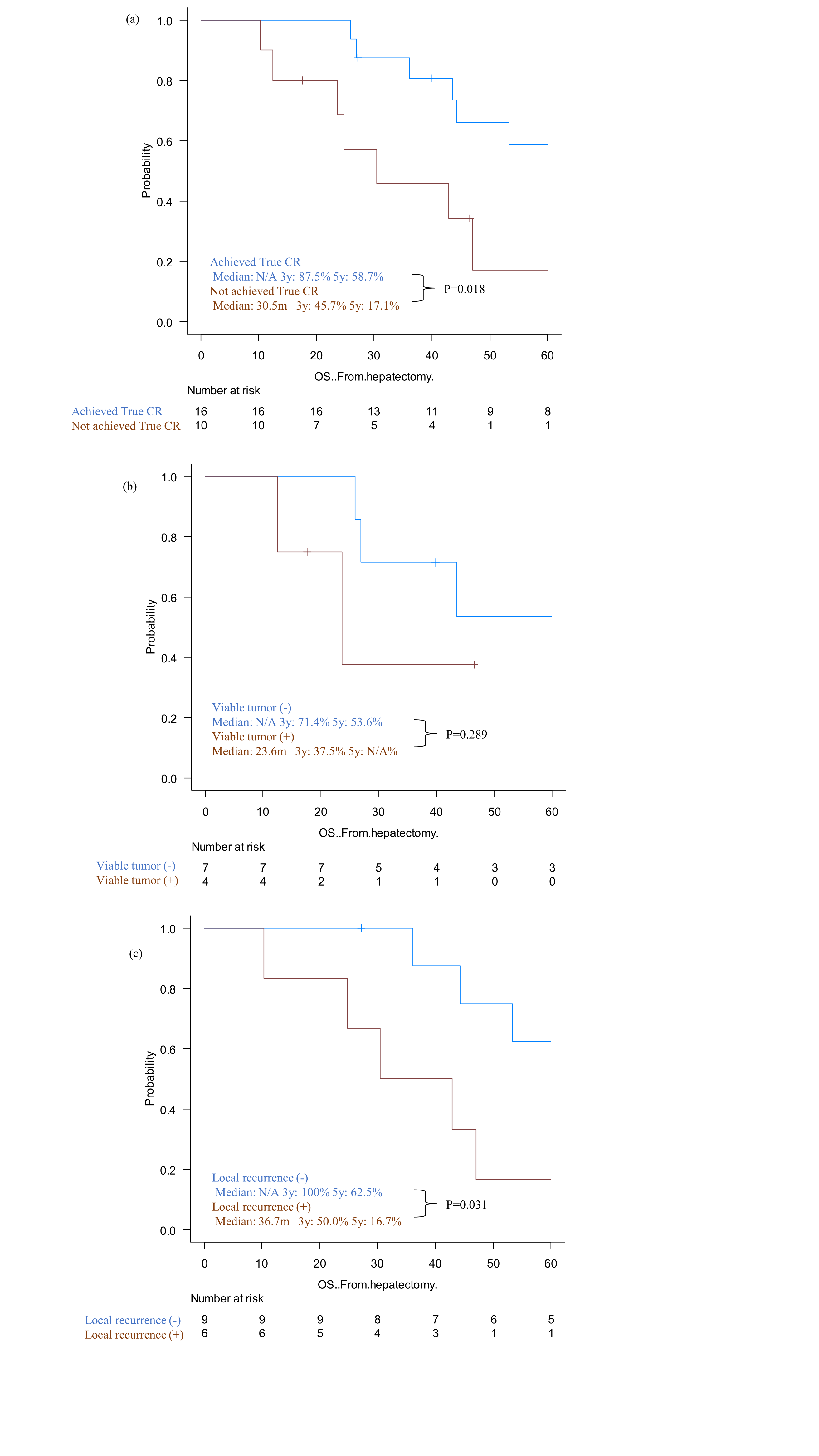

Supplement: Supplementary file 2 — Supplemental FIGURE 2. Comparison of OS according to whether a true CR was achieved. OS was compared among: (a) 26 patients who had DLMs after systemic chemotherapy for initially unresectable CRLMs, (b) 11 patients in whom all DLMs were resected, and (c) 15 patients in whom any DLMs were left unresected. OS, overall survival; CR, complete response; DLMs, disappearing liver metastases; CRLMs, colorectal liver metastases. [file AGS3-10-1239-s002.tif]
